# Sucrosomial^®^ Iron: A New Generation Iron for Improving Oral Supplementation

**DOI:** 10.3390/ph11040097

**Published:** 2018-10-04

**Authors:** Susana Gómez-Ramírez, Elisa Brilli, Germano Tarantino, Manuel Muñoz

**Affiliations:** 1 Department of Internal Medicine, University Hospital Virgen de la Victoria. Campus de Teatinos, 2010 Málaga, Spain; susanagram@yahoo.es; 2 Scientific Department, Alesco S.r.l. Via delle Lenze, 216/B, 56122 Pisa, Italy; elisa.brilli@alescosrl.com; 3 Scientific Department, Pharmanutra S.p.A. Via delle Lenze, 216/B, 56122 Pisa, Italy; g.tarantino@pharmanutra.it; 4 Perioperative Transfusion Medicine, Department of Surgical Specialties, Biochemistry and Immunology, School of Medicine, Campus de Teatinos, 29071 Málaga, Spain

**Keywords:** Anemia, iron deficiency, oral iron salts, intravenous iron, Sucrosomial^®^ iron, M cells, bioavailability, tolerability, efficacy

## Abstract

Iron deficiency (ID) is usually treated with oral iron salts, but up to 50% of patients complain of gastrointestinal side effects, leading to reduced compliance with treatment. Intravenous (IV) iron formulations are increasingly safe, but there is still a risk of infusion, hypersensitivity reactions and the need for venous access and infusion monitoring. Sucrosomial^®^ Iron (SI) is an innovative oral iron formulation in which ferric pyrophosphate is protected by a phospholipid bilayer plus a sucrester matrix (sucrosome), which is absorbed through para-cellular and trans-cellular routes (M cells). This confers SI’s unique structural, physicochemical and pharmacokinetic characteristics, together with its high iron bioavailability and excellent gastrointestinal tolerance. The analysis of the available evidence supports oral SI iron as a valid option for ID treatment, which is more efficacious and tolerable than oral iron salts. SI has also demonstrated a similar effectiveness, with lower risks, in patients usually receiving IV iron (e.g., chronic kidney disease, cancer, bariatric surgery). Thus, oral SI emerges as a valuable first option for treating ID, especially for subjects with intolerance to iron salts or those for whom iron salts are inefficacious. Moreover, SI should also be considered as an alternative to IV iron for initial and/or maintenance treatment in different patient populations.

## 1. Introduction

Data from 187 countries from 2010 revealed that anemia affected up to one-third of the global population, though prevalence varied widely across regions, and iron deficiency (ID) was responsible for about 50% of anemia cases [1]. In a systematic analysis in the Global Burden of Disease Study 2016, iron-deficiency anemia (IDA) was the fourth leading cause of years lived with disability, especially in women [2]. Thus, prophylaxis and management of ID is a first order public issue. The main causes of ID are increased demands, reduced absorption and/or increased loss of iron [3,4] (Table 1). 

**Figure 1:**
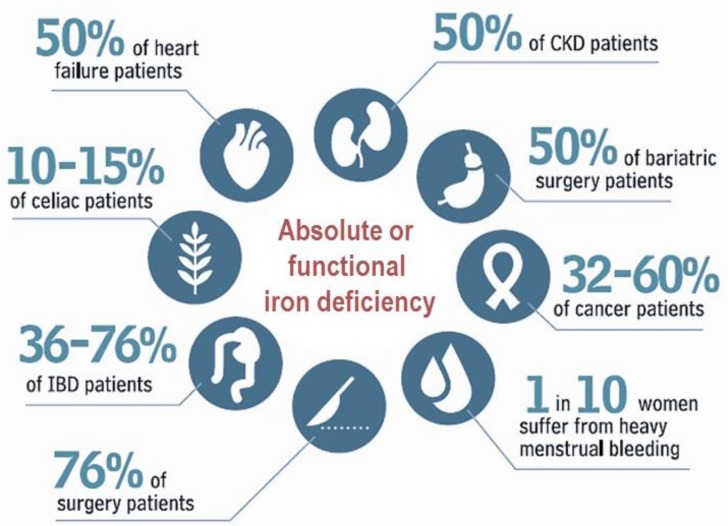
Prevalence of iron deficiency across pathologies. (Data taken from references [5,6,7,8,9,10,11,12,13]).

Nevertheless, the prevalence and consequences of ID may also vary depending on the clinical setting considered [5,6,7,8,9,10,11,12,13] (Figure 1). Following the diagnosis of ID, it is especially relevant to find and address the underlying cause, especially in unexplained and/or recurrent cases, as well as to choose the therapeutic option that safely meets the patient’s needs [14,15,16,17].

## 2. Diagnosis of Iron Deficiency

A correct diagnosis of ID is essential for a safe treatment, but it is sometime elusive. Importantly, the absence of anemia does not exclude ID, because a normal individual must lose most of his iron stores before the hemoglobin (Hb) can fall to values defined by World Health Organization (WHO) as anemia (Hb < 12 g/dL for women and Hb < 13 g/dL for men). In fact, the WHO declares that “mild anemia” is a misnomer, as ID could be well advanced and causes clinical symptoms before Hb reaches the threshold for anemia [18]. The role of non-anemic ID, as a disease looking for recognition, has recently been reviewed: ID is the disease, and anemia is just one of its consequences [17,19].

A patient’s history (including signs and symptoms of ID and co-morbidities) and previous iron supplementation provide some clues. In individuals without anemia, chronic fatigue is the most important symptom (iron is needed for the enzymes involved in energy production). However, generally, clinicians will not relate chronic fatigue to ID. As a result, ID without anemia is almost invariably a casual laboratory finding [20]. 

**Figure 2:**
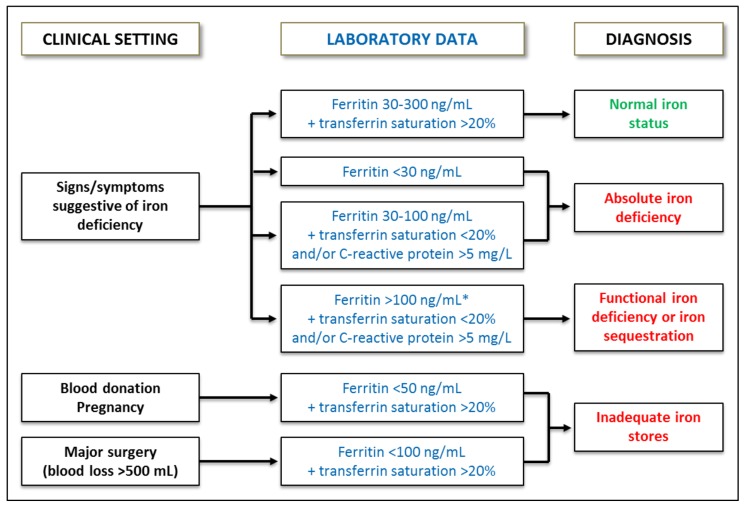
Laboratory assessment of iron status. * Low reticulocyte Hb content (<28 pg), increased hypochromic red cells (>5%) or high soluble transferrin receptor to log ferritin ratio (>2) could identify a component of an absolute iron deficiency in the presence of an inflammation-induced high ferritin level.

In patients whose Hb level is within the normal range, ID should be suspected if a low mean corpuscular Hb (MCH; normal range 28–35 pg) or an increased red cell distribution width (RDW, normal range 11–15) is present [4,21]. The most accurate definition of true ID is a serum ferritin concentration < 30 ng/mL (sensitivity 92%, specificity 98%), though lower values are used in many laboratories [22] (Figure 2). A serum ferritin < 100 ng/mL with a transferrin saturation (TSAT) < 20% is also indicative of ID, especially in the presence of inflammation (Figure 2). In contrast, serum ferritin > 100 ng/mL with a TSAT < 20% usually indicates iron sequestration (also referred to as functional iron deficiency, FID). Treatment with erythropoiesis-stimulating agents (ESA) may also result in FID, as mobilization from stores may be not rapid enough to meet the increased bone marrow demands on iron (Figure 2) [4,21,23]. This provides the basis for iron supplementation in most patients receiving ESA treatment. 

However, as it is an acute phase reactant, high ferritin levels do not exclude ID in patients presenting with an inflammatory status. In these cases, other parameters, such as a low reticulocyte Hb content (<28 pg), increased hypochromic red cells (>5%) or a high soluble transferrin receptor to log ferritin ratio (>2), indicate a component of true ID. Should this be present, iron supplementation may be beneficial [4,14,21].

## 3. Treatment Options for Iron Deficiency 

In addition to searching and addressing the underlying cause, if possible, ID could be treated with oral iron, intravenous (IV) iron and/or blood transfusion, depending on the patient’s Hb levels, tolerance and co-morbidity. Whether it is a new onset, recurrent, explained or unexplained should also be considered for choosing among the different ID treatment options

### 3.1. Oral Iron Supplementation

Oral iron supplements, provided as ferrous or ferric salts, are usually the first line of treatment for uncomplicated ID, because of their availability, ease of administration, and relatively low cost [14,15]. Oral iron has usually been prescribed at a high dose (100–200 mg elemental iron), to be taken 1–3 times a day. However, the bioavailability is 10% to 15% for ferrous iron preparations (sulfate, gluconate, fumarate, etc.), and it is even lower for ferric iron salts or ferric iron complexes (amino acids, polysaccharide, ovo-albumin, etc.). The co-administration of other drugs, such as proton pump inhibitors or antacids, or meals, and the presence of an inflammatory status may further hamper the absorption of oral iron salts [24]. This may prolong the duration of treatment or even render it ineffective [24]. In addition, up to 50% of patients on oral iron (depending on the iron formulation) report gastrointestinal side effects due to the direct toxicity of ionic iron, which may lead to reduced tolerance and adherence to iron supplementation [25,26]. 

Single low doses of iron supplements (40–60 mg/day) are associated with less gastrointestinal side effects and lower hepcidin secretion, resulting in better treatment compliance and enhanced fractional absorption [27,28]. In a randomized study, 90 octogenarian patients with IDA received 15 mg, 50 mg or 150 mg of elemental iron per day. After two months, there were no between-group differences in the levels of Hb (mean increase 1.4 g/dL in all groups) or ferritin, but the adverse effects were significantly more common with higher doses [29]. Therefore, a low single daily dose (40–60 mg) and/or single alternate day dose (80–100 mg) are preferred in order to reduce the side effects and maximize fractional absorption [27,28,29]. Though not formally proven, this emerges as a new paradigm for oral iron supplementation in ID treatment [21].

### 3.2. Intravenous Iron Supplementation

Should the patient develop intolerance to one oral iron salt, or it was proven inefficacious, switching to another oral iron formulation or to intravenous (IV) iron may be appropriate [15]. Different IV iron formulations have been made commercially available for clinical use, such as ferric gluconate (FG), iron sucrose (IS), low molecular weight iron dextran (LMWID), ferric carboxymaltose (FCM), ferumoxytol (FXT), or iron isomaltoside 1000 (ISM). All of them have been shown to have a dose-dependent efficacy for correcting ID [17,24]. However, “newer” IV iron formulations, such as FCM or ISM, which allow for a short-time (15–60 min) infusion of high iron doses (1000 mg or more), are preferred by both physicians and patients, compared to “older” IV formulations [17,24]. 

Nevertheless, though increasingly safer, IV iron formulations are more expensive than oral iron and still carry the need for venous access (side effects at the injection site may occur) and infusion monitoring (there is still a risk of infusion and hypersensitivity reactions) [30]. In this regard, the European Medicines Agency states that “IV iron products should be administered only when staff is trained to evaluate and manage anaphylactic reactions, and resuscitation facilities are immediately available” [30]. In addition, except for the chronic kidney disease population [7], data on the long-term safety of IV iron are scant [24].

### 3.3. Red Blood Cell Transfusion

A patient presenting with severe IDA and alarming symptoms (e.g., hemodynamic instability) and/or risk criteria (e.g., coronary heart disease) should be treated with red blood cell transfusion, using the minimal amount necessary to achieve clinical stability. Adhering to patient-adapted restrictive transfusion criteria and transfusing one unit at the time, with post-transfusion reassessment, is strongly recommended by most guidelines [6,31,32,33,34].

Red blood cell transfusion produces a rapid, albeit transient, rise in Hb, thus increasing oxygen-carrying capacity. However, severe IDA will recur unless the underlying cause is identified and addressed. After hemodynamic stability has been achieved by red blood cell transfusion, additional iron supplementation should be considered [17].

## 4. Sucrosomial^®^ Iron: Preclinical Data

### 4.1. Composition and Structure 

Commonly used oral iron salts are poorly absorbed, with unabsorbed iron leading to gastrointestinal side effects [25]. Newer oral iron supplements have been formulated to increase their tolerability [26]. However, there was still a need for new carriers that not only protect the iron but also enhance its intestinal absorption, thus reducing dosage and side effects [35].

Sucrester is a surfactant derived from the esterification of fatty acids with sucrose (sucrose esters) and has recently been shown to behave as an absorption enhancer because of its ability to reduce intestinal barrier resistance, thus facilitating the passage through para-cellular and trans-cellular routes [36,37]. Sucrester effects depend on both the hydrophilic-lipophilic balance and the fatty acid chain length; therefore, the choice of the appropriate raw material is crucial for developing a formulation with absorption enhancer properties. While there is evidence of the enhancer properties of sucrose esters for the accumulation of drugs in CACO-2 cells [38] and for intestinal permeability in animals [39], its use in oral medicinal product administration has been scarcely studied. 

**Figure 3:**
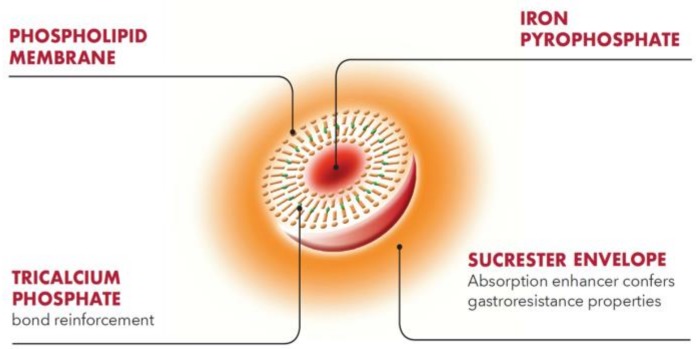
Schematic structure of Sucrosomial^®^ iron.

Sucrosomial^®^ Iron (SI), developed by Alesco srl (Pisa, Italy), represents an innovative oral iron-containing carrier, in which ferric pyrophosphate is protected by a phospholipid bilayer membrane, mainly from sunflower lecithin, plus a sucrester matrix. Further stability and coating are obtained by adding other ingredients (tricalcium phosphate, starch), forming the “sucrosome” and allowing SI to be gastro-resistant and carried through the intestinal tract, without side effects from the interaction between iron and intestinal mucosa (Figure 3). To date, in vitro studies have shown that SI is mostly absorbed as a vesicle-like structure, bypassing the conventional iron absorption pathway. Due to its behavior in the gastrointestinal tract, SI is well tolerated and highly bioavailable, compared to conventional iron salts [40].

### 4.2. Gastro-Resistance and Intestinal Absorption

**Figure 4:**
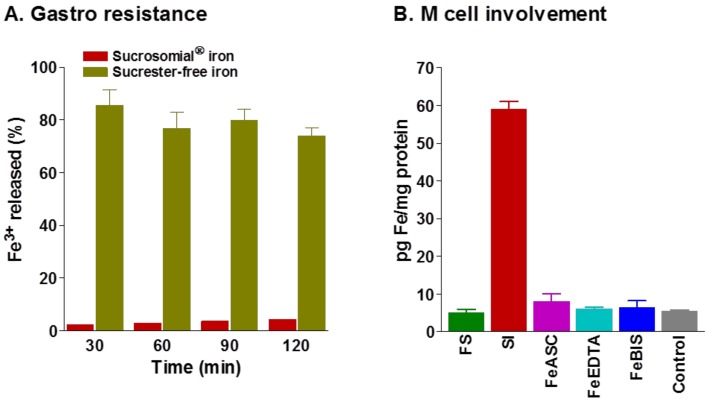
**Gastro-resistance and intestinal absorption of Sucrosomial^®^ iron**. (**A**) Gastro-resistant properties of Sucrosomial^®^ iron compared to a sucrester-free iron preparation in an in-vitro simulated gastric fluid digestion at pH 1.2. (**B**) The involvement of M cells in Sucrosomial^®^ iron uptake was evaluated using an in vitro CACO2/RajiB co-culture. The iron to protein ratio was significantly increased in co-culture cells treated with Sucrosomial^®^ iron (SI), compared to other oral iron formulations: ferrous sulfate (FS), ferrous ascorbate (FeASC), ferrous ethylene-diamine-tetra-acetate (FeEDTA), ferrous bisglycinate (FeBIS), and control (no iron) (data are mean ± SEM, * *p* < 0.05) (Adapted from references [41,42]).

The presence of sucrester confers gastro-resistance properties to SI [41], as demonstrated in *in vitro* studies using a simulated gastric fluid digestion (pH 1.2). After different digestion times (30 to 120 min), the release of ferric iron (III^+^) from SI was very low (<5%), compared to that of a sucrester-free iron preparation (75–85%) (Figure 4A) [41].

Gastro-resistance allows the intact sucrosomes to reach the intestinal mucosa, where they are absorbed. Polled data from several studies indicate the presence of different pathways involved in SI absorption. Ex-vivo permeation experiments, carried out using the excised rat intestine model, have shown that the presence of sucrester protects trivalent pyrophosphate iron in SI against enzymatic reduction and promotes its absorption across the intestinal epithelium through a DMT-1 independent pathway, as it is not affected by BPDS activity (bathophenanthroline disulfonic acid, a divalent iron chelator) [40]. 

The presence of the phospholipids and the sucrester matrix allows the pyrophosphate iron in SI to be absorbed as a vesicle-like structure through para-cellular and trans-cellular routes. *In vitro* experiments using the MatTek EpiIntestinal human 3D tissue model have confirmed the presence of vesicle-like structures during the intestinal absorption of SI and its different absorption kinetics, compared to ferrous sulfate (FS) and ferrous bisglycinate (FeBIS) [42]. Over time, a greater increase of iron concentration in the basolateral compartment was observed in tissues treated with SI (2.7 ± 1.7 g/mg protein), compared to samples treated with FS (1.3 ± 1.1 g/mg protein) and FeBIS (1.6 ± 1.1 g/mg protein), indicating an endocytosis-mediated cellular uptake, which was confirmed by transmission electron microscopy analysis [42].

Microfold cells of the Peyer’s patches (M cells) are involved in the transfer of particles and microbes from the luminal side of the intestine to the lamina propria, where they are presented to immune cells. M cells have been shown to provide a pathway for delivering orally administered vesicle-like particles to the lymphatic system [43,44]. However, the transfer efficacy of this pathway has also been shown to be greatly influenced by the physicochemical properties of the transported particles [43,44]. The possible role of an M cell-mediated pathway in SI absorption was investigated using an in vitro CACO2/RajiB co-culture system. Experimental data show that the presence of M cells (RajiB cells) increased the absorption of SI but not that of conventional oral iron salts, such as FS or FeBIS (Figure 4B). This evidence confirms that M cells can support the intestinal absorption of SI. In ex-vivo experiments using isolate rat intestine and fluorescein, labeled SI, it has been demonstrated that, after passing through M cells, SI was taken up by CD68+ macrophages [42].

### 4.3. Bioavailability

**Figure 5:**
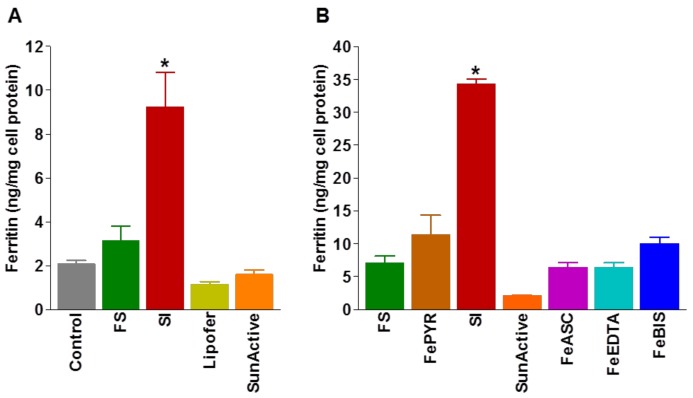
**Bioavailability experiments on CACO-2 cells**. Ferritin expression by cells treated with Sucrosomial^®^ iron (SI) was significantly increased, compared to cells treated with ferrous sulfate (FS), phospholipid containing ferric pyrophosphate (Lipofer^®^), or micronized, dispersible ferric pyrophosphate (SunActive^®^) **(A)** or different iron salts, FS, ferric pyrophosphate (FePYR), ferrous ascorbate (FeASC), ferrous ethylene-diamine-tetra-acetate (FeEDTA), ferrous bisglycinate (FeBIS), and control (no iron) **(B)** (Data are mean ± SEM, * *p* < 0.001 SI vs. other iron compounds) (Adapted from references [41,45]).

Most probably, the involvement of different cellular routes in SI absorption underlies its high bioavailability, and this may explain its efficacy in improving hemoglobin and ferritin concentrations. Data from CACO-2 cell cultures show that the administration of SI increases 3-fold ferritin accumulation, compared to ferrous sulfate, and 3.5-fold, compared to phospholipid containing ferric pyrophosphate (Lipofer^®^) or micronized, dispersible ferric pyrophosphate (SunActive^®^) (Figure 5A), indicating that the SI technology increases ferritin iron accumulation within enterocytes [45]. Furthermore, in vitro experiments, comparing SI with commercially available iron salts, show that SI was able to significantly increase the ferritin concentration in CACO-2 cells, compared to tested iron salts (Figure 5B) [41]. 

**Figure 6:**
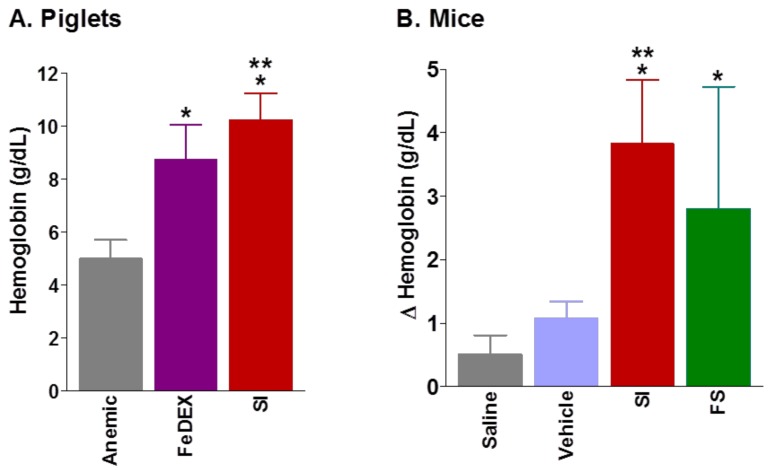
**Iron supplementation in anemic piglets and mice**. **(A)** Hemoglobin concentration in anemic piglets treated with iron dextran (FeDEX) or Sucrosomial^®^ iron (SI) (Data are mean ± SD; * *p* < 0.001, treatment vs. anemic; ** *p* < 0.001, SI vs. FeDEX). **(B)** Change in hemoglobin levels in anemic mice after a 14-day treatment with Sucrosomial^®^ iron (SI) or ferrous sulfate (FS). (Data are mean ± SD; * *p* < 0.01, SI or FS vs. saline; ** *p* < 0.05, FS vs. vehicle) (Adapted from references [46,47]).

Data from cell cultures show that SI was able to increase the ferritin expression in enterocytes in vitro, but this evidence was not sufficient to demonstrate the high bioavailability of SI in vivo. Therefore, SI bioavailability was subsequently investigated in iron deficient new-born piglets and mice. In piglets, a 4-week course of oral SI supplementation efficiently prevented the deterioration of the hematological status and contributed to the recovery from IDA, as shown by the significant increase in Hb concentration, compared to iron dextran treated animals. In addition, oral SI supplementation increased duodenal L-ferritin protein levels, compared with animals treated with parenteral iron dextran (Figure 6A) [46]. In anemic mice, treated with iron administered via gavage for 2 or 4 weeks, SI was able to improve the hemoglobin levels and iron status (Figure 6B) [47]. Bioavailability data obtained from animals are interesting, as they indicate that different animals affected by IDA respond to oral SI supplementation in a similar manner and that the efficacy of SI is comparable with all forms of oral iron salts. Remarkably, the efficacy of SI has been demonstrated, not only in animal models of uncomplicated IDA, but also in some clinical conditions, in which the absorption of oral iron is drastically reduced (e.g., celiac disease, post-bariatric surgery, ACI, IRIDA]) [48,49,50,51,52].

### 4.4. Distribution

Usually iron distribution and storage are measured by the quantification of the total iron and ferritin expression in target tissues. Ferritin-bound iron indicates the ability of the cell to internalize and store iron and, indirectly, the absorption of the administered iron. Anemic piglets and mice treated with SI were able to store iron in the ferritin of their spleens and livers. Moreover, a mild but significant increase of serum iron and transferrin saturation was observed in both IDA animal models [46,47].

A bioavailability study was also performed in healthy rats treated with ferric pyrophosphate or SI, in which the concentrations of trivalent iron in blood were measured over time (h). Blood concentrations of trivalent iron were higher in animals treated with SI after the first 3 h. Pharmacokinetic profiles show that the area under the curve (AUC) and maximal plasma concentration of iron (C_max_) for SI were significantly higher than those for ferric pyrophosphate. Furthermore, 5 h after the oral administration, SI, but not ferric pyrophosphate, led to a measurable increase of the trivalent iron content in liver and bone marrow [53]. These data suggest that SI has a greater bioavailability, and that the iron supply which exceeds the requirements for hematopoiesis and metabolic processes, is stored in the hepatocytes [54]. 

### 4.5. Iron Homeostasis

Hepcidin, a 25-amino acid peptide, synthetized by hepatocytes, regulates systemic iron homeostasis, and its levels can be increased in response to inflammation or iron overload [55]. Similarly, oral supplementation with iron salts also induces hepcidin up-regulation, which regulates iron release into the bloodstream and then to target organs [35].

The effects of oral supplementation with ferrous sulfate or SI, provided at the same concentration (1 mg/kg/day), on liver hepcidin mRNA and circulating hepcidin levels were investigated in IDA mice. While SI-treated mice showed a minor, non-significant increase in liver hepcidin mRNA and serum hepcidin levels, both were significantly increased in ferrous sulfate-treated animals. In parallel, FS induced the expression of two inflammatory markers, as well as the suppressor of the cytokine signaling 3 (Socs3) and C-reactive protein (CRP), while SI did not [47]. This suggests that FS supplementation induces hepcidin up-regulation through a double mechanism. First, a direct effect of the absorbed iron on peri-portal hepatocytes was observed in ID women receiving FS at doses of ≥60 mg/day [27]. Secondly, the direct toxicity of non-absorbed iron on intestinal mucosa induces an inflammatory response [56]. In contradistinction, most SI is not released into the portal blood stream, but into the lymphatic circulation (M cells route) and, later, the arterial circulation, before reaching the liver. Moreover, the ferric pyrophosphate in SI does not interact with duodenal mucosa, as it is protected by the sucrosome, and it has also been suggested that the phospholipidic bilayer carriers may also exert anti-inflammatory properties [57]. Such a different behavior of SI could be relevant, since hepcidin reduces the iron availability by inhibiting the cellular iron export. 

## 5. Sucrosomial^®^ Iron for the Management of Iron Deficiency in Different Clinical Settings

As stated above, SI has unique structural, physicochemical and pharmacokinetic characteristics, together with a high iron bioavailability and excellent gastrointestinal tolerance. These properties make SI the most suitable formulation for the oral treatment of ID, even in clinical settings (e.g., CKD, cancer, bariatric surgery, etc.), where IV iron seemed to be the only therapeutic option [48,49,50,51,52]. We will review the efficacy and safety of oral SI for treating ID in the most common clinical scenarios.

### 5.1. Obstetrics

Iron deficiency in pregnancy continues to present a significant health problem throughout the world. There is evidence that ID and IDA are associated with an increased risk of a poor pregnancy outcome (e.g., low birth weight, prematurity), low neonatal iron deposits, preeclampsia and post-partum hemorrhage [5]. In a propensity score analysis (n = 12,470), severe pregnancy anemia was also associated with an increased risk of peri-partum mortality [58]. In the postpartum period, anemia is associated with decreased physical performance, reduced cognitive abilities and impaired lactation [5].

In order to reduce the risk of low birth weight, maternal anemia and iron deficiency, a recent consensus statement recommends daily oral supplementation of 30–60 mg iron and 400 µg folic acid, as part of routine antenatal care (GRADE 1B) [5]. However, compliance with recommended oral iron intake among pregnant women varies, mostly due to the gastrointestinal side effects. Multivitamin and mineral compounds are not the best way for supplementation, mainly when ID or IDA is present, as most of them do not contain adequate amounts of iron, vitamin B_12_, C or D. Indeed, the EMPIRE study in Portugal found that IDA had a very high prevalence among pregnant women (54.2%), despite the fact that over 80% of them were receiving iron supplementation, mostly administered as multivitamin and mineral products [59].

In this regard, a recent study on non-anemic pregnant women, presenting with Hb > 10.5 g/dL at 12–14 weeks of gestation, were randomly assigned to receive no iron (control; n = 20), ferrous iron 30 mg/day (FI; n = 20), SI 14 mg/day (SI-14; n = 20) or SI 28 mg/day (SI-28; n = 20) for up to 6 weeks postpartum (Table 2). Compared to the control and FI groups, the SI-28 group showed significantly higher Hb levels at 28 weeks and in the postpartum period. Ferritin levels at 20 and 28 weeks and at 6 weeks postpartum (*p* < 0.01) were significantly higher in the SI-28 group, compared to the control. Interestingly, fewer women from the SI-28 group developed anemia (10%), compared to the control (30%), FI (25%), and SI-14 (25%). Moreover, no differences in hematological parameters were observed between the group receiving SI 14 mg/day and that receiving FI 30 mg/day, thus demonstrating a higher bioavailability and allowing for the reduction of doses and side effects [60] (Table 2). 

In a series of 148 consecutive deliveries, 8 non-anemic women (mean Hb: 12.1 g/dL), who developed postpartum anemia due to bleeding (mean: 858 mL; range: 700–1600 mL), received SI (60 mg twice daily). After one week, the mean Hb increase was 1.5 g/dL, and no gastrointestinal or systemic side effects were witnessed [61]. 

Thus, published evidence on SI in relation to preventing anemia during pregnancy is promising, and large studies, further evaluating the role of SI in pregnancy and in the postpartum period, would be helpful (Eudra CT: 2017-000994-35).

### 5.2. Oncology 

Both ID and IDA are highly prevalent in onco-hematological patients. IDA and chemotherapy-induced anemia (CIA) are generally treated with red blood cell transfusion, ESAs and/or iron supplementation. However, there is controversy on the safety and cost issues regarding different iron compounds and administration routes [23,62].

A recent position statement recommends investigating the presence of anemia and/or iron deficiency in all cancer patients, but especially in those scheduled for cytotoxic chemotherapy, radiotherapy or surgery. This should be carried out before and during treatment in order to plan the most appropriate therapeutic strategy [23].

According to the most recent ESMO guidelines, patients with CIA (Hb ≤ 11 g/dL or Hb decrease of ≥ 2 g/dL from a baseline level of ≤ 12 g/dL) and absolute ID (serum ferritin < 100 ng/mL) should receive iron treatment to correct ID. In the case of FID (TSAT < 20% and serum ferritin > 100 ng/mL), iron treatment should be administered before the initiation of and/or during ESA therapy (with both the originator and biosimilar products, approved by the EMA), or as mono-therapy [6]. 

Regarding the iron formulation that should be used, ESMO guidelines indicate that oral iron is considered only for patients with both absolute ID and non-inflammatory conditions (CRP < 5 mg/L). Even if ESMO guidelines recommend the use of IV iron in cases of FID, they stated that the long-term safety of IV iron in oncology has not yet fully established [6]. Therefore, SI could be taken into consideration in cases of inflammatory conditions and anemia, as SI absorption does not seem to be affected by hepcidin [23,35].

In several small case-series and pilot studies of anemic oncologic patients, with or without chemotherapy, oral SI (30–60 mg/day for 2–6 months) was shown to increase Hb levels, with very few gastrointestinal side effects (Supplementary material, Table S1). More recently, a retrospective study of patients presenting with moderate CIA (Hb 8–10 g/dL), and no ID or FID, assessed the performance of oral SI (30 mg/day; n = 33) in improving the Hb response to ESA (Darbepoetin 500 μg/3 weeks), compared to IV FG (125 mg/week; n = 31) [51] (Table 2). After 8 weeks of treatment, there were no differences between the groups with respect to Hb response (70% vs. 71%, respectively), as defined by an increment in Hb ≥ 2 g/dL and/or a final Hb ≥ 12 g/dL. There were also no differences in red cell transfusion rates (one patient in each group) or change in the quality of life. Oral SI was well tolerated, with only one patient showing gastrointestinal toxicity, whereas 2 infusion reactions were witnessed with IV FG. 

### 5.3. Nephrology 

Iron deficiency is one of the main causes of anemia in patients with CKD, and iron supplements, along with ESAs, constitute the basis of its treatment, both for patients not on dialysis (ND-CKD) and those who are hemodialysis-dependent (HD-CKD), but disparities exist in the guidelines and position papers for anemia treatment across the world [7,63].

Though mortality and adverse effects rates in CKD patients receiving oral or IV iron supplementation are similar, a meta-analysis (24 studies, 3187 patients) found that hypotensive reactions were more frequent with IV iron, whereas more gastrointestinal adverse events were observed with oral iron [64]. A recent randomized, controlled trial in 128 anemic CDK patients suggested a possible higher incidence of cardiovascular events and hospitalization for infection in the IV iron arm, compared to the oral iron arm [65]. In contrast, a trial evaluating IV versus oral FS in 626 anemic ND-CKD patients, with ID and not receiving ESA therapy, found no difference in the infection rates or cardiac events between patients receiving a higher FCM dose (500–1000 mg/4 weeks), lower FCM dose (200 mg/4 weeks) or oral FS (200 mg/day) during the 56 weeks follow-up (3.9%, 3.3%, and 3.8%, respectively) [66]. It is worth noting that, in this trial, only 21.6% of patients receiving oral iron showed an Hb increase of at least 1 g/dL, and < 30% of early non-responders responded at any subsequent time point during the follow-up, suggesting that the early consideration of alternative therapy would be beneficial in this population [67]. In a cohort of 58,058 HD-CKD patients, IV iron doses greater than 400 mg/month were associated with higher cardiovascular death rates [68]. The conclusion should be that “too much iron is bad” for CKD patients, but further large clinical studies are needed (such as the on-going PIVOTAL trial; EudraCT Number: 2013-002267-25), whereas the oral versus IV administration of iron is an on-going debate [24,63].

In several case series on ND-CKD patients (mostly with mild anemia), oral SI (30 mg/day), with or without ESA, was shown to be efficacious in maintaining and/or increasing Hb, ferritin and TSAT levels during different follow-up periods (from 3 to 24 months), with virtually no gastrointestinal side effects (Supplementary material, Table S2). In a randomized open-label trial, 99 ND-CKD patients with IDA (Hb ≤ 12 g/dL, ferritin ≤ 100 ng/mL, TSAT ≤ 25%) were assigned (2:1) to receive oral SI (30 mg/day) for 3 months or a total dose of 1000 mg of IV FG (125 mg/week), and they were followed-up for 4 months [50]. At the end of the treatment period, the Hb levels were similar in both groups (11.4 g/dL vs. 11.7 g/dL, respectively), though the replenishment of iron stores was greater in the IV FG group (ferritin 86 ng/mL vs. 239 ng/mL, respectively; *p* < 0.05) (Table 2). Hb concentrations decreased more rapidly after iron withdrawal, although significantly fewer adverse events were observed in the oral group (*p* < 0.001), and adherence to treatment was similar in the two groups. Therefore, this study shows that short-term, low-dose oral SI is as efficacious as IV FG for correcting anemia in ND-CKD patients and suggests that there is no risk of iron overload during its long-term use. Similar results were observed in two preliminary studies comparing oral IV and IV FG, with or without ESA, in 34 anemic HD-CKD patients (Supplementary material, Table S2). 

Recently, in 3 CKD patient populations (pre-dialysis, peritoneal dialysis, and post-transplant), who did not respond to conventional oral iron supplementation, Darbá et al. [69] assessed the economic impact of switching from intravenous iron (FCM or IS) to SI. Using a 4-year budget impact model (2017–2020), the progressive increase of SI use (up to 10% of market shares) would lead to over €750,000 savings. 

### 5.4. Gastroenterology

Anemia is the most frequent systemic complication in *inflammatory bowel disease* (IBD), *celiac disease* (CD), *non-celiac gluten sensitivity* (NCGS) and *autoimmune atrophic gastritis* (AAG) [70]. In IBD patients, anemia is more frequent among those with Crohn’s disease, though its prevalence in studies varies according to the definition criteria, type of patients, and year of publication, but ID and ACD are the most common causes [9]. 

Oral iron supplementation in IBD patients may result in worsening disease symptoms (flares), which can be attributed to iron-induced oxidative stress, but also to microbiota alterations. After 3 months of iron supplementation, shifts in gut bacterial diversity and composition were found in IBD patients, with oral FS (120 mg of elemental iron per day) differentially affecting bacterial phylotypes and fecal metabolites, compared with IV iron sucrose therapy (3–4 doses of 300 mg) [71]. However, in an experimental model of colitis in mice (induced by the administration of dextran sodium sulfate [DSS]), the effect of dietary iron supplementation (500 mg/kg) on survival depended on the formulation used, and this was either beneficial (FeBIS) or highly detrimental (ferric ethylene-diamine-tetra-acetic acid), most likely due to the modulation of the microbiota [72]. In addition, a hem-enriched intestinal lumen (due to a hem-rich diet, hem-based iron supplementation or intestinal bleeding) led to changes in bacterial flora composition (with a decrease in *Proteobacteria* and a reduction of *Firmicutes)*, which were similar to, though less pronounced than, those observed after DSS administration [72]. Therefore, oral iron supplementation in IBD patients with ID is challenging, and guidelines are prone to recommend the use of IV iron [9]. However, initial clinical data in IBD patients suggest that oral iron formulations with improved tolerability, such as SI or ferric maltol, may represent a viable alternative to IV iron [73].

In several case series of IBD patients with mild-to-moderate IDA (n = 92, including 46 intolerant to ferrous sulfate [74]), SI (30–60 mg/day for 2–3 months) has been shown to be efficacious in rising Hb concentrations (+0.92 g/dL), as well as ferritin and TSAT levels, with very few gastrointestinal side effects (Table 2) (Supplementary material, Table S3). In comparative 2–3 month treatment courses, the efficacy of SI (30–60 mg/day; n = 38) in increasing Hb was higher than that of FS (105–210 mg/day, n = 14) (mean Hb change +2.7 g/dL vs. 1.4 g/dL, respectively), despite lower elemental doses (Supplementary material, Table S3), and similar to that of IV iron sucrose (100 mg/session, up to 500–1000 mg) (mean Hb change: +1.7 g/dL vs. +1.8 g/dL, respectively) [75] (Table 2).

Celiac disease is a common intestinal autoimmune pathology, which presents with laboratory abnormalities, irritable bowel syndrome, osteopenia, fertility problems and iron deficiency [76]. A prospective study evaluated the efficacy of a 3-month course of SI supplementation (30 mg/day) in anemic patients with CD, who could not tolerate oral FS (n = 24) (NCT02916654) [48]. Additionally, naïve patients were assigned to receive oral FS (105 mg/day) (n = 19). After a 3-month follow-up, a significant improvement in all iron parameters was observed in both groups. Both treatments increased Hb levels, compared to the baseline, with a similar proportion of patients presenting Hb values within the normal range (70% vs. 82%, respectively; *p* = ns), although the elemental iron dose with SI was one-third of that with FS (Table 2). As evaluated by visual analog scale (VAS) scores, patients receiving SI reported a lower severity of abdominal symptoms and a higher increase in general well-being, compared to those receiving FS [48]. 

Sucrosomial^®^ iron (30 mg/day for 15 days, plus 15 mg/day for 75 days) has also been shown to be effective in treating IDA, in a series of non-CD gluten-sensitivity patients (n = 28), and in increasing Hb (+2.8 g/dL) and ferritin levels (+11 ng/mL) (Supplementary material, Table S3).

Autoimmune atrophic gastritis is another autoimmune entity, which may be triggered by *Helicobacter pylori* infection, in which autoantibodies against gastric parietal cells and/or an intrinsic factor are characteristically present. Mucosal atrophy leads to hypo- or achlorhydria, and most AAG patients develop anemia, either due to cobalamin deficiency (older patients) or ID (younger patients) [70]. Importantly, AAG is responsible for 20–27% of IDA cases, which are refractory to oral iron supplements, and use to be treated with IV iron [77].

To assess the efficacy of oral SI, 20 consecutive AAG women (100% with gastric parietal cells autoantibodies, 20% with intrinsic factor autoantibodies) with recently diagnosed IDA (Hb < 120 g/dL) were enrolled in a prospective observational study [78]. Patients received SI (120 mg/daily, either fasting or during meals) for 8 weeks. Only 3 patients dropped out due to intolerance (2) or lack of compliance (1). Compared to the baseline values, after 8 weeks, there were significant increments in Hb (from 10.5 g/dL to 12.5 g/dL), ferritin (from 7 ng/mL to 27 ng/mL) and TSAT (from 8% to 18%) (Table 2).

Bariatric surgery, especially the malabsorptive procedure, can be associated with a risk of nutritional deficiency, including iron, which increases over the years, and women of childbearing age are the most vulnerable group [10]. In a 4-year follow-up, after gastric bypass or sleeve gastrectomy procedures at a single institution (n = 353, 73% women), the investigators found that ID prevalence was significantly reduced in men (17.2% vs. 40%), but significantly increased in women (31.5% vs. 26.1%), especially in those of childbearing age (<50 years) [79]. In these settings, traditional oral iron formulations present clear and significant limitations regarding tolerance and efficacy, and patients use to be switched to IV iron.

A case-control study included 40 women of childbearing age, who were receiving IV iron sucrose supplementation after bariatric surgery (300 mg every 3 months). Of these women, 20 were switched to oral SI (28 mg/day for 3 months), while another 20 received a dose of IV iron sucrose (300 mg). Hemoglobin, ferritin, and TSAT levels were measured, before and three months after the treatment switch, and no between-group differences were found [49] (Table 2). Thus, for patients developing ID after bariatric surgery and requiring IV iron, oral SI could be an alternative maintenance therapy. 

### 5.5. Cardiology 

As stated above, iron is needed for proteins and enzymes involved in oxygen transport (hemoglobin), storage (myoglobin), and utilization for energy production (respiratory chain) in skeletal and cardiac muscle cells. ID, with or without anemia, affects 50% of congestive heart failure (CHF) patients and is independently associated with a reduced physical performance, decreased quality of life and increased risk of mortality [8]. Treatment of ID, as defined by serum ferritin <100 ng/mL or ferritin between 100 and 299 ng/mL and TSAT <20%, in patients with chronic heart failure, is strongly recommended by the European Society of Cardiology [80]. 

The mechanisms of ID in heart failure are still not well understood. Chronic heart failure is considered to have a low-grade inflammatory status, which increases the circulating levels of hepcidin. In turn, hepcidin binds ferroportin, especially at the enterocytes, promoting its internalization and degradation, thus preventing iron absorption, while iron recirculation from macrophages seems to be less affected [4,14,55]. The alteration in the composition of the intestinal microbiota, known as intestinal dysbiosis, may also contribute to the perpetuatation of the inflammatory status [35]. A high prevalence of malnutrition and reduced iron absorption due to intestinal edema could also be involved [35]. In a recent randomized controlled trial in CHF patients (the IRONOUT study), oral iron polysaccharide (150 mg, bid) was demonstrated to be inefficacious for correcting ID [81]. In contrast, the European guidelines recommend the administration of IV iron for treating ID in this patient population [80]. 

However, a prospective pilot study has evaluated the possible role of oral SI supplementation in 30 patients with CHF and ID iron deficiency, with or without anemia [82]. Twenty patients received oral SI (30 mg/day for 3 months) and 10 served as controls (no iron). All were on stable, evidence-based medical therapy for at least 1 month, and there were no differences in baseline clinical and laboratory parameters between groups. At 3 months, SI treatment improved iron parameters, while Hb levels remained stable. There was also an improvement in quality of life, as assessed by Kansas City Cardiomyopathy Questionnaire (from 55.7 to 61.8; *p* = 0.038), and a trend towards a longer 6-min walked distance (from 318 m to 332 m; *p* = 0.065) and lower B-natriuretic peptide (from 643 to 535; *p* = 0.360). All patients in the SI group adhered to the protocol, and no side effects were witnessed. No change in any of the assessed parameters was observed in the control group. These results are in line with those from 3 small case-series (n = 29) (Supplementary material, Table S4), but large, confirmatory studies are needed. In this regard, 2 randomized controlled trials, comparing oral SI with oral ferrous bisglycinate or a placebo (PREFER-HF study), or oral SI with IV FCM (IVOFER-HF study), are currently on-going.

### 5.6. Internal Medicine 

Anemia is a frequent condition among hospitalized surgical and critically ill patients, compromising their clinical outcome. However, the role of anemia, as a risk factor for a poor outcome in other hospitalized patients, has hardly been investigated. In a random sample of patients, admitted to the internal medicine ward in 2015, the prevalence (53%) and severity (46% moderate, 7% severe) of anemia was high and most probably caused by chronic inflammation, blood loss, and/or hemodilution [83]. It seemed to be linked to older age, a higher Charlson comorbidity index, a longer length of hospital stay and increased in-hospital mortality, but it was underdiagnosed and undertreated [83]. On admission to the internal medicine ward, data from 771 consecutive patients revealed that 67% presented with anemia, which was associated with an increased risk of in-hospital mortality (RR 1.82, 95% CI 1.21–2.74) [84]. Iron deficiency (58%), with (41%) or without (18%) anemia, was also highly prevalent [84]. Therefore, an appropriate anemia management protocol for this patient population should be established, including an appropriate provision of iron supplementation. 

Patients with *myelodysplasia* (MSD) frequently exhibit anemia with FID, for which IV iron may be effective [6]. However, preliminary data suggested that oral SI may be as effective as IV iron in MSD patients (Supplementary material, Table S5). More recently, the efficacy of SI (28 mg/day) in supporting the erythropoietic response to an originator (group A) or biosimilar (group B) epoetin-α was studied in 92 MSD patients with refractory anemia. Patients also received vitamin B_12_ (400 mg/day orally) and calcium levofolinate (7.5 mg/day orally) to avoid deficiencies of maturation factors [85]. Responder rates (as defined by an Hb increment of ≥ 1.5 g/dL after 3 months of epoetin treatment) were similar in both groups (50% and 43%, respectively) and higher than that reported in the literature [86], thus suggesting the efficacy of oral SI supplementation. 

In a small sample of young women with chronic inflammatory anemia due to *autoimmune diseases* (systemic erythematosus lupus, rheumatic fibromyalgia, connectivitis), the efficacy SI (60 mg/day, n = 9) was compared to that of FS (210 mg/day) over a 3-month course [86]. There were no differences in the baseline Hb (8.5 g/dL vs. 9.0 g/dL, respectively), iron status (%TSAT, Ferritin) or inflammatory markers (CRP). Compared to FS, SI resulted in significant improvements in Hb (11.5 g/dL vs. 9.5 g/dL, respectively) and ferritin (260 ng/mL vs. 100 ng/mL, respectively). Additionally, SI, but not FS, was associated with a significant reduction in ESR and CRP levels [87].

*Bleeding* is also a common cause of anemia in the internal medicine ward, with red blood cell transfusion being the default treatment, but in many cases we could care for patients without exclusively resorting to blood components. A recent study included 90 patients with moderate-to-severe IDA due to non-neoplastic gastrointestinal or gynecologic bleeding, without inflammation but with intolerance/refractoriness to FS [88]. Patients were randomized to receive a high dose of SI (120 mg/day for one month; SI group; n = 45), with or without food or antacid therapy, or IV FG (62.5 mg/day until cover total ID; FG group; n = 45). There were no differences in the baseline Hb concentration (8.5 g/dL vs. 8.2 g/dL, for SI and FG groups, respectively), and both treatments were equally effective in increasing the Hb (12.0 g/dL vs. 12.5 g/dL, after 4 weeks, respectively) (Table 2), though treatment costs were significantly lower for the oral SI (120 €/month) than for the IV ferric gluconate (300 €/month). Adverse drug events were observed in 12 (26%) patients from the SI group (epigastric pain, diarrhea) and in 10 (22%) from the FG group (hypotension, urticarial, headache), but no patient required transfusion (Table 2). These data seem to confirm those obtained with SI supplementation in several case series and observational studies of patients with IDA of different origins (mostly bleeding) (Supplementary material, Table S5).

**Figure 7:**
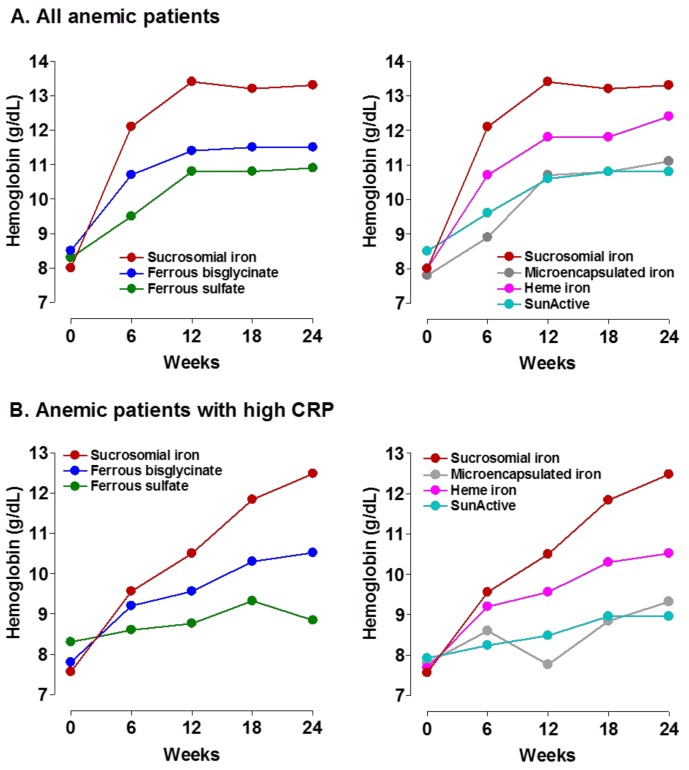
Comparative efficacy of different oral iron formulations for treating bleeding-induced moderate-to-severe anemia. SunActive^®^, micronized ferric pyrophosphate. Each oral iron formulation was tested in 60 patients (Data taken from reference [89]).

In another multicenter study, 300 patients with moderate-to severe IDA (Hb < 11 g/dL, ferritin < 30 ng/mL) due to gastric (44%) or intestinal (56%) bleeding were randomized 1:1:1:1:1:1 to receive 60 mg of elemental iron daily of oral FS, microencapsulated iron (Saccarate iron), micronized ferric pyrophosphate (SunActive^®^), SI, heminic bisglycinated iron, or bisglycinated iron. Patients’ characteristics and follow-up times (12–24 weeks) were similar in all six groups. Compared to any other oral iron formulation tested, SI led to consistently higher Hb increments from week 6, both for the whole study population (Figure 7A) and for the subgroup of patients presenting with inflammation (high CRP) (Figure 7B). At week 24, mean Hb concentrations in SI-treated patients were 13.2 g/dL for the whole, and 12.5 g/dL for the high CRP subgroup. Gastrointestinal side effect rates were low with all formulations (6–12%), except for FS (30%). Therefore, among the different oral iron formulations tested in this patient population, SI showed the fastest and greatest efficacy in correcting IDA, which was more evident in patients presenting with high CRP values [89].

### 5.7. Surgery

Pre-operative anemia is frequent among patients scheduled for major elective surgery (30–40%) [13], being an independent risk factor for a poor outcome (increased rates of morbidity, mortality and readmission) and prolonged length of hospital stay [12], but enhancing the deleterious effects of blood loss and red cell transfusion. Postoperative anemia is even more frequent, affecting up to 80–90% of patients [90].

Absolute iron deficiency and iron sequestration are the leading causes of preoperative anemia (70% of cases), whereas surgery-associated blood loss and inflammation may induce and/or maintain postoperative anemia [13]. Hematinic deficiencies without anemia are also frequent and may hamper pre-operative Hb optimization and/or recovery from postoperative anemia. 

As modifiable risk factors, preoperative anemia (Hb < 13 for both genders) and hematinic deficiencies should be detected, classified and treated prior to any major surgery [21]. However, the role of preoperative oral iron supplementation in treating ID, FID or IDA in these patient populations has been scarcely investigated, while available evidence indicated that it is not useful in the postoperative period [21,24,90].

A retrospective study evaluated the efficacy of preoperative SI in 200 paired-matched patients undergoing prosthetic hip surgery (2106) in terms of blood transfusion requirements, length of hospital stay and postoperative Hb recovery [91]. Preoperative iron supplementation with SI (30 mg/day for 3–4 weeks, preoperatively) was offered to 100 patients with Hb 12–13.5 g/dL, for women, or 13–14 g/dL, for men, and ferritin < 100 ng/mL (ID) or ferritin > 100 ng/mL, if there was an elevated CRP or TSAT < 20% (FID). Another 100 patients with the same demographic and laboratory characteristics, who had not received SI, served as a control group. Compared to the control group (no iron supplementation), SI supplementation led to a reduction in the number of transfused units (0 units vs. 7 units, respectively) and the length of the hospital stay (4 days vs. 6.5 days, respectively), with an estimated cost saving of 1763 €/patient. Additionally, higher Hb levels were observed in the SI group, 30 days after discharge (13.4 ± 1.5 vs. 10.2 ± 1.2, respectively). Obviously, a confirmatory randomized control trial on the beneficial effects of SI supplementation is warranted.

In this regard, the CardioSideral Heart Surgery (NCT03560687), a prospective study in 1000 consecutive patients undergoing heart surgery, randomized to either SI supplementation or no treatment (control), is on-going. Its main outcome variable is the reduction in transfusion rates, but the changes in Hb and iron parameters, number of transfused units, postoperative quality of life (6-min walk test), tolerability and cost-effectiveness of SI will also be assessed. 

## 6. Efficacy of Sucrosomial® Iron: An Overview

Most relevant evidence on the bioavailability, tolerability and efficacy of oral SI in different preclinical and clinical settings has been presented as lectures or communications in the 3rd, 4th, 5th and 6th International Multidisciplinary Courses on Iron Anemia and other international meetings. However, a growing number of studies have been already published as full peer-review papers [40,42,47,48,49,50,51,60,69,85,91]. Characteristics and results for a number of clinical studies are summarized in Table 2.

Preclinical studies clearly demonstrated that SI has unique structural, physicochemical and pharmacokinetic characteristics. The presence of sucrester confers gastro-resistance to SI, protects its trivalent pyrophosphate iron against enzymatic reduction, and promotes its absorption across the intestinal epithelium by a DMT-1 independent pathway, which is greatly mediated by M cells. All this enables oral SI to have a high iron bioavailability and a low gastrointestinal toxicity. 

The analysis of available clinical evidence seems to support oral SI as a new valid opportunity for iron supplementation, which is more comfortable, efficacious (lower doses, higher Hb increments and/or better replenishment of iron stores) and tolerable than traditional oral iron salts (Table 2). Sucrosomial iron has been also demonstrated to have a similar effectiveness, with lower risks, in clinical settings where IV iron was the usual treatment option (e.g., CKD, cancer, bariatric surgery, etc.) (Table 2). 

Thus, the administration of oral SI emerges as a valuable first option for treating uncomplicated iron deficiency, especially for subjects with intolerance to iron salts or those for whom iron salts are inefficacious. Moreover, oral SI should also be considered as an alternative to IV iron for initial and/or maintenance treatment in different patient populations. Nevertheless, appropriately sized randomized control trials are needed to confirm the promising results obtained with oral SI supplementation in different clinical settings. 

## Figures and Tables

**Table 1:** Main causes of iron deficiency.

*Increased demands:* ▪ Body growth (infancy and childhood) ▪ Pregnancy and lactation ▪ Recovery from blood loss ▪ Treatment with erythropoiesis stimulating agents *Limited external supply or absorption* ▪ Poor intake ▪ Inappropriate diet with deficit in bioavailable iron and/or ascorbic acid (including excess of dietary fiber, phenolic compounds from tea or coffee, and soya products) ▪ Malabsorption (autoimmune atrophic gastritis, gastric resection, bariatric surgery, inflammatory bowel disease, celiac disease, non-celiac gluten sensitivity, *Helicobater pylori* infection) ▪ Medications (AntiH_2_, PPI, antacids, etc.) ▪ Increased hepcidin levels (e.g., IRIDA or ACI) ▪ Molecular defects in iron transport proteins (e.g., heme oxygenase or DMT1 deficiencies) *Increased iron losses:* ▪ Bleeding trauma ▪ Gastrointestinal bleeding (peptic ulceration, neoplasia, inflammatory bowel disease, vascular malformations, medications [anti-inflammatory, anti-platelet or anticoagulant agents]) ▪ Genitourinary bleeding ▪ Menses and multi-parity ▪ Multiple diagnostic phlebotomies (medical “vampirism”) ▪ Blood donation ▪ Dialysis (particularly hemodialysis)

ACI, anaemia of chronic inflammation; AntiH_2_, histamine H_2_ receptor antagonists; DMT1, divalent metal transporter 1; IRIDA, iron-refractory iron deficiency anemia; PPI, proton pump inhibitors.

**Table 2:** Efficacy and tolerance of oral Sucrosomial^®^ iron in different clinical setting (9 studies, 513 patients).

Author [ref] (year) Study Type	Study Population	Treatment Compound (Dose) Duration	Baseline Hb (g/dL)	Final Hb (g/dL)	Baseline Ferritin (ng/mL)	Final Ferritin (ng/mL	Baseline TSAT (%)	Final TSAT (%)	GI Side Effects
Parisi et al. [60] (2017) RCT	80 non-anemic pregnant women 12–14 week	Control, no iron (n = 20) FS (30 mg/day) (n = 20) SI (14 mg/day) (n = 20) SI (28 mg/day) (n = 20) Up to postpartum week 6	12.0 11.9 12.0 11.9	11.6 11.8 12.0 12.0	47 44 52 53	31 43 41 50	28 27 28 27	26 27 30 29	
Mafodda et al. [51] (2017) RCT pilot	64 patients with solid tumor	SI (30 mg/day) + DEPO (500 mcg/3 weeks) FG (125 mg/wk IV) + DEPO (500 mcg/3 weeks) 2 months	9.4 9.2	12.7 12.9	---	---	---	---	3% 0%
Pisani et al. [50] (2014) RCT	99 patients with chronic kidney disease	SI (30 mg/day) (n = 66) FG (125 mg/week IV, TID: 1000 mg) (n = 33) 3 months	10.8 10.7	11.4 11.7	71 68	86 239	16.5 17.0	18.3 21.5	12% 18%
Bastida et al. [74] (2016) Case series	46 patients with inflammatory bowel disease intolerant to FS	SI (30 mg/day) 3 months	11.2	11.8 *	14.3	16.0	8.7	16.2	11%
Stuklov et al. [75] (2018) Observational	40 patients with inflammatory bowel disease	SI (60 mg/day) (n = 25) IS (100 mg/session, 500–1000 mg) (n = 15) 3 months	10.1 10.0	11.8 11.8	---	---	---	---	No
Elli et al. [48] (2016) Observational	34 patients with celiac disease	SI (30 mg/day) intolerant to FS (n = 18) FS (105 mg/day) (n = 16) 3 months	10.0 10.0	12.1 12.3	12 (all)	---	11 (all)	---	Not stated
Farinati et al. [78] (2018) Case series	20 patients with autoimmune atrophic gastritis	SI (120 mg/daily, either fasting or during meals) 8 weeks	10.5	12.5	7	27	8	18	10%
Ciudín et al. [49] (2017) Case-control	40 women after bariatric surgery	SI (28 mg/day) (n = 20) IVI (Iron sucrose 300 mg) (n = 20) 3 months	12.4 12.5	12.3 12.7	102 98	89 96	22.9 23.6	24.1 26.3	0% 0%
Giordano et al. [87] (2016) RCT	90 patient with IDA due to bleeding	SI (120 mg/day) (n = 45) FG (62.5 mg/day IV to cover TID) (n = 45) 4 weeks	8.5 8.3	12.0 12.5	5 7	---	---	---	26% 22% **

DEPO, darbepoetin; FG, ferric gluconate; FS, ferrous sulphate; GI, gastrointestinal; IBD, inflammatory bowel disease; IDA, iron deficiency anemia; IV, intravenous; PCI, percutaneous coronary intervention; RCT, randomized controlled trial; SI, Sucrosomial^®^ iron; TID, total iron deficiency; TSAT, transferrin saturation. * Recovery from IDA: 25.7%, improvement on quality of life (EuroQoL) form 60.9 at baseline to 65.5 at the end of study period; ** Hypotension, urticaria, headache.

## Supplementary Materials

The following are available online at http://www.mdpi.com/1424-8247/11/4/97/s1, Table S1: Sucrosomial^®^ iron (SI) administration in oncologic patients (10 studies, 241 patients), Table S2: Sucrosomial^®^ iron (SI) administration in patients with chronic kidney disease (CKD) (11 studies, 294 patients), Table S3: Sucrosomial^®^ iron (SI) administration in patients with gastrointestinal disease (7 studies, 122 patients), Table S4: Sucrosomial^®^ iron (SI) administration in cardiology patients (8 studies, 161 patients), Table S5: Sucrosomial^®^ iron (SI) administration in Internal Medicine (10 studies, 236 patients).

## Abbreviations

AAG, autoimmune atrophic gastritis; ACI, anemia of chronic inflammation; AntiH2, histamine H2 receptor antagonists; AUC, area under the curve; BPDS, bathophenanthroline disulfonic acid; CACO2, human colon cancer cell line; CD, celiac disease; CHF, congestive heart failure; CIA, chemotherapy-induced anemia; CKD, chronic kidney disease; Cmax, maximal plasma concentration; CRP, C-reactive protein; DEPO, darbepoetin; DMT-1, divalent metal transporter-1; DSS, dextran sodium sulfate; ESA, erythropoiesis stimulating agent; FCM, ferric carboxymaltose; FeASC, ferrous ascorbate; FeBIS, ferrous bisglycinate; FeDEX, iron dextran; FeEDTA, ferrous ethylene-diamine-tetra-acetate; FePYR, ferric pyrophosphate; FG, ferric gluconate; FI, ferrous iron; FID, functional iron deficiency; FS, ferrous sulfate; FXT, ferumoxytol;GI, gastrointestinal; Hb, hemoglobin; HD-CKD, hemodialysis-dependent CKD; IBD, inflammatory bowel disease; ID, iron deficiency; IDA, iron deficiency anemia; IRIDA, iron-refractory iron deficiency anaemia; IS, iron sucrose; ISM, iron isomaltoside 1000; IV, intravenous; LMWID, low molecular weight iron dextran; M cell, microfold cell of Peyer’s patches (RajiB cell); MCH, mean corpuscular hemoglobin; MCV, mean corpuscular volume; MSD, myelodysplasia; NCGS, non-celiac gluten sensitivity; ND-CKD, CKD non on dialysis; PCI, percutaneous coronary intervention; PPI, proton pump inhibitors; RCT, randomized controlled trial; SI, Sucrosomial^®^ iron; Socs3, suppressor of cytokine signaling 3; TID, total iron deficiency; TSAT, transferrin saturation; VAS, visual analog scale; WHO, World Health Organization.
