# Supplementary material for: Sucrosomial® Iron: A New Generation Iron for Improving Oral Supplementation
Source: Pharmaceuticals (Basel). 2018 Oct 4;11(4):97. doi: 10.3390/ph11040097 (PMC6316120; doi:10.3390/ph11040097)
Supplement: Supplementary file 1 [file pharmaceuticals-11-00097-s001.pdf]

Table S1. Sucrosomial iron (SI) administration in oncologic patients (10 studies, 241 patients)

Author (year) [ref] Study type	Patients	Treatment Compound (Dose) Duration	Baseline Hb (g/dL)	Final Hb (g/dL)	Baseline Ferritin (ng/mL)	Final Ferritin (ng/mL	Baseline TSAT (%)	Final TSAT (%)	GI side effects
Renso et al (2015) [1] Case series	12 patients with lymphoproliferati-ve disease	SI (30 mg/day) +DEPO 150 mcg/w Chemotherapy 2 months	10.1	10.9	---	---	---	----	4%
Petrungaro et al. (2015) [2] Case series	10 patients with lymphoma (4 HL,6NHL)	SI (30 mg/day) After chemotherapy 2 months	10.0	11.2 (↑QoL)	43	93	---	---	---
Grillone et al. (2016) [3] Case series	30 patients with solid tumors	SI (30 mg/day) Chemotherapy 2 months*	11.2	11.3					10%
Romano et al. (2016) [4] Case series	25 Hodgkin lymphoma ≥2B	SI (30 mg/day) Chemotherapy End of treatment**	10.2	12.8	90	277	14.3	35.9	No
Barni et al. (2017) [5] Case series	16 patients with solid tumor	SI (30 mg/day) Palliative chemotherapy 3 months**	11.2	10.9	---	---	13.5	20.6	No
Sabbatini et al (2017) [6] Case series	30 patients wih solid tumors	SI (30 mg/day) (n=15)<br>SI (60 mg/day) (n=15)<br>3 months**	10.5<br>9.8	12.0<br>12.0	---	---	---	---	Some dyspepsia and diarrhea
Poyato et al. (2017) [7] Case series	9 patients with solid tumors	SI (30 mg/day) Chemotherapy 2 months***	9.8	10.7	---	---	---	---	11%
Monari et al (2016) [8] Observational	15 Advanced prostate cancer with bone metastases	SI (30mg/day) (n=7)<br>No iron (n=8)<br>Chemo-radiotherapy 6 months	11.1<br>10.9	12.2<br>9.7	---	---	---	---	--<br>Well tolerated
Barragans et al. (2016) [9] RCT pilot	15 patients with peritoneal carcinomatosis	SI (30mg/day) (n=8)<br>FS (80 mg/day) (n=7)<br>3 months	10.4<br>9.5	12.5<br>11.9	529<br>1048	---	10<br>8	---	25% mild<br>29% mild
Barzaghi et al. (2016) [10] Observational	15 patients advanced rectal cancer & bleeding	SI (30 mg/day) (n=11)<br>FH (?)+folic acid<br>14 days	8.0	11.6<br>11.4	100	---	25	---	No

*2 patients started ESA; **No ESA or blood transfusion during study period, 70% reached delta Hb ≥2 g/dL or Hb ≥12 g/dL; *** one patient transfused. DEPO, darbepoetin; ESA, erythropoiesis stimulating agent; FH, ferrum Haussman?; GI, gastrointestinal; QoL, quality of life; TSAT, transferrin saturation.

References:

1. Renso R, Bolis S, Casaroli I, et al. Use of darbopoietin and oral liposomal iron (Sideral Forte) in chemotherapy associated anemia in patients with lymphoproliferative disease. Expert Review of Hematology, 2015; 8 (Suppl 1):S24
2. Petrungaro A, Mineo G, Cingari R, et al. Benefits assessment of liposomal iron (Sideral® Forte) administration to hematologic patients in follow-up after chemotherapy. Expert Review of Hematology, 2015; 8 (Suppl 1):S18-S19
3. Grillone F, Gualteri S, Ventura M, et al. Efficacy and tolerability of Sucrosomial® Iron in elderly (≥75 years) patients with solid tumors treated with anticancer agents: a retrospective analysis. Expert Review of Hematology, 2016; 9 (Suppl 1): 28.
4. Romano A, Conticello C, Motta G, et al. Oral Sucrosomial® Iron supplementation in patients affected by Hodgkin lymphoma with mild anemia before chemotherapy: an observational study. Expert Review of Hematology, 2016; 9 (Suppl 1): 30-31.
5. Barni S, Lonati V, Ghilardi M, et al. Sucrosomial® iron for transfusion prevention in cancer patients on chemotherapy. Expert Review of Hematology, 2017; 10 (Suppl 1): 23-24.
6. Sabbatini G, Bonfitto F. Effectiveness of two different dosage regimes of Sideral® forte in anemic patients with solid tumor. Expert Review of Hematology, 2017; 10 (Suppl 1): 24-25.
7. Poyato E, Villacé P, Pujades C, et al. Safety and efficacy of Sucrosomial® iron in cancer patients with anemia. Expert Review of Hematology, 2017; 10 (Suppl 1): 26-27.
8. Monari F, Morgantin AG, Frezza G, et al. Oral Sucrosomial® Iron (Sideral® Forte) supplementation in patients with advanced prostate cancer and bone metastasis treated with 223 radium dichloride. Expert Review of Hematology, 2016; 9 (Suppl 1): 24-25.
9. Barragans M, Camblor M, Cuerda C, et al. Sucrosomial® Iron versus ferrous sulfate for anemia in patients undergoing peritoneal carcinomatosis with cytoreductive surgery and hyperthermic intraperitoneal chemotherapy. Expert Review of Hematology, 2016; 9 (Suppl 1): 22-23.
10. Barzaghi D, Cristiano O, Rlmo M, Guida C. Sucrosomial® Iron and radiotherapy in the neoadjuvant treatment of rectal cancers. Good news for patients? Expert Review of Hematology, 2016; 9 (Suppl 1): 26-27.

Table S2. Sucrosomial iron (SI) administration in patients with chronic kidney disease (CKD) (11 studies, 294 patients)

Author<br>(year) [Ref]<br>Study type	Patients	Treatment<br>Compound (Dose)<br>Duration	Baseline<br>Hb<br>(g/dL)	Final<br>Hb<br>(g/dL)	Baseline<br>Ferritin<br>(ng/mL)	Final<br>Ferritin<br>(ng/mL	Baseline<br>TSAT<br>(%)	Final<br>TSAT<br>(%)	GI<br>side<br>effects
Cuzzola et al.<br>(2016) [1]<br>Case series	35 ND-CKD<br>Intolerant<br>to FS	SI (30 mg/day)?<br>3 months	9.3	11.1	---	---	11.2	8.9	No
Dimokvic et al.<br>(2016) [2]<br>Case series	31 ND-CKD3-4	SI (30 mg/day)<br>ESA (no change in dosage)<br>6 months	10.2	10.3	213	169	26.8	24.4	Mild*
Arenas et al.<br>(2016) [3]<br>Case series	24 ND-CKD3-4	SI (30 mg/day)<br>6 months	11.1	12.8	34	75	13.8	26.1	No
Arrizabalaga et al.<br>(2017) [4]<br>Case series	31 ND-CKD3<br>71% intolerant<br>to conventional oral<br>iron	SI (30 mg/day)<br>12 months	12.0	11.9	100	116	17.0	18.7	3%
Griveas et al.<br>(2017) [5]<br>Case series	30 ND-CKD3-5	SI (30 mg/day)?<br>18 months	11.0	11.9	43	99	---	---	No
Griveas et al.<br>(2018) [6]<br>Case series	40 ND-CKD3-5	SI (30 mg/day)?<br>24 months	11.6	12.0	74	66	---	---	No
Equitani et al.<br>(2016) [7]<br>Observational	16 ND-CKD<br>Severe anemia	SI (60mg/day)+ ESA (n=8)<br>No iron + ESA (n=8)<br>3 months	8.6<br>8.9	12.6<br>11.4	12<br>21	68<br>21	24<br>28	39<br>19	NO
Moussa-Abdi et al<br>(2015) [8]<br>Observational	28 ND-CKD	SI (60/mg/day) (n=14)<br>FS (100 mg/day) (n=14)<br>3 months	11.2<br>11.3	11.7<br>11.4	78<br>182	90<br>228	17.9<br>22.3	22.4<br>31.8	14%<br>58%
Panichi et al.[#]<br>(2015) [9]<br>RCT	12 HD-CKD	SI (30-180 mg/week)<br>FG (30-180 mg/week)<br>3 months	12.7<br>12.0	12.7<br>12.6	---<br>---	---<br>---	24.0<br>27.6	21.0<br>30.8	No
Pistoni et al.<br>(2016) [10]<br>RCT?	22 HD-CKD	SI (360 mg/week)+ESA (n=13)**<br>FG (16-190 mg/week)+ESA (n=9)***<br>3 months	10.7<br>11.1	11.4<br>11.4	312<br>285	177<br>250	21.6<br>18.6	20<br>15.7	---
Cucchiari et al.<br>(2018) [11]<br>Case series	25 HD-CKD	SI (90 mg/week)<br>3 months<br>All patients were previously receiving IV ferric gluconate (62.5 mg/week)	11.2	11.0	226	97	30	16	No

ND, not on dialysis; HD, hemodialysis; FG, ferric gluconate; ESA, erythropoiesis stimulating agent; EPO, erythropoietin; TSAT, transferrin saturation index; GI, gastro-intestinal.
*More frequently mild dyspeptic symptom and less frequently constipation. [#]Need for EPO 36% with SI vs. 57% with FG. **EPO dose decreased by 2000 IU/week in 38% and increased in 23% of SI patients. ***EPO dose increased by 2000 IU/week in 67% and decreased in 11% of FG patients.

References:

1. Cuzzola C, Mancini A, Giancaspro V. Anemia in chronic kidney disease patients: comparison between Sucrosomial® iron and ferrous sulfate. Expert Review of Hematology 2016; 9 (Suppl 1): 33.
2. Dimkovic N, Maslarevic Radovic V, Jankovic A, et al. Is liposomal iron good alternative over IV iron for maintenance therapy in CKD patients [SP319]. Nephrology Dialysis Transplantation 2016; 31 (Suppl 1): i193–i199,
3. Arenas MD, Herrera AC, Chacón A, Alzate E. Efficacy, tolerance, and adherence to treatment with Sucrosomial® Iron in patients with chronic kidney disease stages 3–4 and iron deficiency. Expert Review of Hematology 2016; 9 (Suppl 1): 34-35.
4. Arrizabalaga P. Oral iron treatment with sucrosomial iron in patients with CKD and anemia. V International Multidisciplinary Course on Iron Anemia, Florence, April 2017.
5. Griveas I. Efficacy and tolerability of oral Sucrosomial® iron in CKD patients with anemia. Expert Review of Hematology 2017; 10 (Suppl 1): 8-10.
6. Griveas I. Efficacy and tolerability of oral Sucrosomial® iron in CKD patients with anemia and its association with CKD progression parameters. VI International Multidisciplinary Course on Iron Anemia, Lisbon, April 2018.
7. Equitani F. Erythropoietin (EPO) plus oral Sucrosomial® Iron versus EPO alone for the treatment of severe anemia in no end-stage chronic kidney disease. Expert Review of Hematology 2016; 9 (Suppl 1): 35-36.
8. Moussa-Abdi F, Alvaredo de Beas F, Velasco –Pilar R, et al. Hierro liposómico oral en ERCA. Experiencia con dos dosis diarias y comparación con sulfato ferroso (P.188). Nefrología 2015; 35 (suppl 1):54
9. Panichi V, Scatena A, Digiorgio A, Paoletti S. A randomized trial investigating the effects of oral liposomal iron (Sideral Forte) versus intravenous iron gluconate in CKD hemodialysis patients. Expert Review of Hematology 2015;8 (Suppl 1):19
10. Pistoni G, Di Martino M, Veziano E, Saffioti S. Effectiveness of Sucrosomial® Iron (Sideral® Forte) in dialysis patients in therapy with intravenous iron and erythropoietin (EPO). 57º Congresso Nazionale Societá Italiana di Nefrologia, Milano, October 2016
11. Cucchiari D. Oral Sucrosomial® iron versus intravenous gluconate iron in hemodialysis pateinmts: Focus on Hb maintenance and oxidative stress. VI International Multidisciplinary Course on Iron Anemia, Lisbon, April 2018.

Table S3. Sucrosomial iron (SI) administration in patients with gastrointestinal disease (7 studies, 122 patients)

Author (year) [Ref] Study type	Patients	Treatment Compound (Dose) Duration	Baseline Hb (g/dL)	Final Hb (g/dL)	Baseline Ferritin (ng/mL)	Final Ferritin (ng/mL	Baseline TSAT (%)	Final TSAT (%)	GI side effects
Inflammatory Bowel Disease (IBD)									
Scarpulla et al. (2016) [1] Case series	10 IBD	SI (30 mg/day) 2 months	10.5	12.3	≤200	---	≤20	---	No
Stuhlov et al (2017) [2] Case series	6 IBD	SI (60 mg/day) 3 months	11.1	12.4	12.4	20.2	8.1	15.7	No
Indriolo et al. (2014) [3] Observational	27 IBD	SI (30 mg/day) (n=7)<br>FS (105 mg/day) (n=8)<br>No iron (n=11)<br>3 months	10.6<br>10.9<br>11.4	12.6<br>12.3<br>11.9	---	---	---	---	7.1%<br>12.5%
Romano et al. (2016) [4] Observational	12 IBD	SI (60 mg/day) (n=6)<br>FS (210 mg/day) (n=6)<br>2 months	8.0<br>8.0	11.5<br>9.5	5<br>6	15<br>9	---	---	50% mild<br>100%
Celiac disease /gluten-sensitivity									
Ragozzino et al (2015) [5] Case series	6 Celiac disease<br>28 Non-celiac gluten sensitivity	SI (30 mg/day 15d, 15 mg/day 75d)<br>3 months	8.8<br>9.7	11.5<br>12.5	13<br>18	23<br>29	---	---	---
Scorsone et al. (2015) [6] Observational	24 Celiac disease<br>All T1DM	SI (30 mg/day) (n=12)<br>FS (105 mg/day) (n=12)<br>1 month	?<br>?	+1.27<br>+0.82	---	---	19.1<br>17.4	23.6<br>18.7	---
Bariatric surgery									
Badiali et al (2017) [7] Case series	9 Bariatric surgery<br>RYGBP	SI (30-60 mg/day)<br>3 months	11.4	12.6	6.7	19.0	---	---	No

FS, ferrous sulphate; GI, gastro-intestinal; IS, IV iron sucrose; TSAT, transferrin saturation index; T1DM, type 1 diabetes mellitus

References:

1. Scarpulla et al. Effectiveness and compliance of oral Sucrosomial® Iron (Sideral® Forte) in asymptomatic inflammatory bowel disease. Exp Rev Hematol 2016; 9 (Suppl 1): 32.
2. Stuklov NI, Basiladze IG, Pivnih AV, et al. Sideral® Forte – the first experience of 3 month therapy of anemia in inflammatory bowel diseases. Exp Rev Hematol 2017; 10 (Suppl 1): 19-21.
3. Indriolo A, Signorelli S, Greco S, Ravelli P. Comparison between liposomial iron and ferrous sulfate in patients with iron anemia deficiency and inflammatory bowel disease, 20º Congresso Nazionales delle Malattie Digestive, Napoli, March 2014.
4. Romano M. Sucrosomial® Iron is effective in correcting inflammatory bowel disease anemia and is more tolerable than sulfate iron. Exp Rev Hematol 2016; 9 (Suppl 1): 32-33.
5. Ragozzino g, Riccio A, Mattera E. Effectiveness of oral liposomal iron (Sideral Forte) in patients with intestinal malabsorption (Celiac disease and gluten sensitivity). Exp Rev Hematol 2015; 8 (Suppl 1): 21-22.
6. Scorsone A, Calandrino C, Ferranti R, et al. Iron supplementation with liposomal formulation (Sideral Forte) in celiac patients with iron deficiency and Type 1 diabetes mellitus. Exp Rev Hematol 2015; 8 (Suppl 1): 19-20.
7. Badiali S, Mozzi E. A protocol for the treatment of iron anemia after bariatric surgery with Sucrosomial® iron. Exp Rev Hematol 2017; 10 (Suppl 1): 21-22.

Table S4. Sucrosomial iron (SI) administration in cardiology patients (8 studies, 161 patients)

Author (year)[Ref] Study type	Patients	Treatment Compound (Dose) Duration	Baseline Hb (g/dL)	Final Hb (g/dL)	Baseline Ferritin (ng/mL)	Final Ferritin (ng/mL	Baseline TSAT (%)	Final TSAT (%)	GI side effects
Congestive heart failure									
Marazia et al (2017)[1] Case series	9 patients with CHF-LVDF (EF ≤39%)	SI (60 mg/day) * 1 month	10.3	11.0	32	67	---	----	---
Putorti et al. (2017)[2] Case series	10 patients with Hypertensive heart disease	SI (60 mg/day) 5 weeks	10.5	11.5	---	---	---	---	No
Karavidas et al. (2017)[3] Case series	10 patients with CHF-LVDF (EF ≤39%)	SI (30 mg/day)** 3 months	12.8	13.4	48	102	---	---	No
Cardiac surgery									
Testa et al. (2017)[4] Case series	28 patients after cardiac surgery	SI (30 mg/day, months 1 and 3)*** 3 months	10.0	11.9	334	63	---	---	No?
Grossi et al. (2017)[5] Case series	16 patients after cardiac surgery	SI (120 mg/day, 7 days)<br>SI (60 mg/day, 14 days)<br>2-3 weeks[#]	9.7<br>10.0	10.2<br>11.0	---	---	---	---	No
Buioni et al. (2017)[6] Case series	22 patients after cardiac surgery	SI (120 mg/day) 3 weeks	10.0	12	---	---	---	---	No
Other interventions									
Ruperto et al. (2017)[7] Observational	50 patients percutaneous coronary intervention	SI (30 mg/day) (n=25)<br>FS (105 mg/day) (n=25)<br>3 months post-PCI	8.9<br>9.1	11.2<br>11.0	---	---	---	---	0%<br>32%
Pagliani et al. (2017)[8] Observational	16 patients in cardiac rehabilitation	SI (60 -120 mg/day) (n=8)<br>FS (105 mg/day) (n=8)<br>14 days	9.5<br>9.2	10.1<br>9.9	500<br>600	400<br>870	---	---	0%<br>33%

*BNP and CRP decreased with treatment; **BNP and CRP decreased with treatment, 6MWD increased after treatment, and NYHA improved; ***BNP and CRP decreased with treatment, 6MWD increased after treatment; [#]6MWD increased after treatment.

References:

1. Marazia S, Loderi S, Magliari F, et al. Sucrosomial® iron supplementation can be a useful support treatment in patients with heart failure and anemia. Exp Rev Hematol 2017; 10 (Suppl 1): 33.
2. Putorti G. Benefits of Sucrosomial® oral iron in patients with heart disease and concomitant iron-deficiency anemia. Exp Rev Hematol 2017; 10 (Suppl 1): 37.
3. Karavidas A, Trokanis E, Farmakis D, et al. Oral Sucrosomial® iron in heart failure patients with iron deficiency. Exp Rev Hematol 2017; 10 (Suppl 1): 12-13.
4. Testa R, Venturini E, Sansoni C, et al. Oral Sucrosomial® Iron in post cardiac surgery patients. Efficacy and tolerability in a follow up of 3 months. Exp Rev Hematol 2017; 10 (Suppl 1): 12-13.
5. Grossi A, Baldari F, Codraro S, et al. Sucrosomial® iron: short-term efficacy compared to administration of 4 capsules/day for 7 days vs. 2 capsules/day for 14 days in post cardiac surgery patients for myocardial revascularization. Exp Rev Hematol 2017; 10 (Suppl 1): 31.
6. Buioni D, Nardella S, Maselli D. Effect of Sucrosomial® iron in early time after cardiac surgery. Exp Rev Hematol 2017; 10 (Suppl 1): 36-37.
7. Ruperto C, Ricca G, Antonio AA, et al. Oral Sucrosomial® iron supplementation in patients underwent percutaneous coronary intervention: safety, efficacy and tolerability. Exp Rev Hematol 2017; 10 (Suppl 1): 33-34.
8. Pagliani L, Payadattil S, Marigo L, et al. Hospital protocol for evaluating effectiveness and timing of use of Sucrosomial® iron in cardiac rehabilitation departments. Exp Rev Hematol 2017; 10 (Suppl 1): 35-36.

Table S5. Sucrosomial iron (SI) administration in Internal Medicine (10 studies, 236 patients)

Author (year)[Ref] Study type	Patients	Treatment Compound (Dose) Duration	Baseline Hb (g/dL)	Final Hb (g/dL)	Baseline Ferritin (ng/mL)	Final Ferritin (ng/mL	Baseline TSAT (%)	Final TSAT (%)	GI side effects
Alimenti et al (2015)[1] Case series	30 IDA various origin	SI (30 mg/day)? 2month	10.2	??	---	---	---	---	10%
Campanella et al (2015)[2] Case series	16 IDA various origin	SI (30 mg/day) 40 days<br>SI (30 mg/day) 60 days	10.0<br>10.8	11.9<br>12.6	<20<br><20	----	----	---	No
Scifo et al (2015)[3] Case series	9 hemorrhoidal disease with IDA	SI (60 mg/day) 3 month	9.4	10.8	10	80	18	34	No
Vallerio et al (2016)[4] Case series	8 HVC-related cirrhosis	SI (60 mg/day)* 1 month	9.4	10.1	10	36	19	24	10%
Nasuti et al (2016)[5] Case series	30 IDA various origin	SI (60 mg/day) 2 month	9.8	12.1	---	---	---	---	10%
Nadir et al (2017)[6] Case series	4 IDA because of bleeding	SI (300 mg/day, 10 days + 120 mg/day, 50 days) 2 month	9.0	12.5	14.5	103	---	---	No
Svanera et al (2017)[7] Case series	3 IDA various origin	SI (30 mg/day) 4 month	7.3	11.3	1.6	87	---	---	No
Berardi et al. (2015)[8] RCT pilot	20 MDS with refractory anemia	SI (14mg/day) + EPO 40,000 IU/week (n=10)<br>No iron + EPO 40,000 IU/week (n=10)<br>3 months	8.8<br>9.0	12.5<br>11.5	---	---	<20<br><20	---	No
Parisi et al. (2016)[9] Observational	34 patients with systemic sclerosis	SI (60 mg/day) (n=21)**<br>FS (105 mg/day) (n=22)<br>3 months	10.2<br>10.7	13.4<br>11.9	130<br>110	240<br>150	---	---	0%<br>23%
Bellodi et al. (2016)[10] Observational	82 patients with IDA	SI (30 mg/day)<br>FG or FCM (500 mg) + SI (30 mg/day)<br>6-7 months	10.3<br>8.8	11.6<br>12.2	7.5<br>5	27.5<br>27	---	---	3.7%

EPO, recombinant erythropoietin; FCM, ferric carboxymaltose; FG, ferric gluconate; FS, ferrous sulphate; GI, gastrointestinal; Hb, haemoglobin; IDA, iron deficiency anaemia; TSAT, transferrin saturation.

*Reduction of aortic stiffness. ** SI treatment reduced ESR and CRP levels.

References

1. Alimenti M, Della Vida GL, Cipriani L. Tolerability of dietary supplementation with protected liposomal iron (Sideral) in elderly patients with complex clinical and under polypharmacy treatment suffering from iron deficiency anemia of various origins. Exp Rev Hematol 2015; 8 (Suppl 1): S25.
2. Campanella MP, Ebbli A, Gaibazzi D, et al. Effectiveness and compliance of oral liposomal iron (Sideral Forte) treatment for iron deficiency anemia: a valid alternative to iv iron therapies. Exp Rev Hematol 2015; 8 (Suppl 1): S31-S32.
3. Scifo M. Liposomal iron and ascorbic acid (Sideral Forte) supplementation in the treatment of iron deficiency anemia in patients with hemorrhoidal disease. Exp Rev Hematol 2015; 8 (Suppl 1): S21.
4. Vallerio P, Stucchi M, SiricoD, et al. Sucrosomial® Iron and aortic stiffness in cirrhotic patients. Exp Rev Hematol 2016; 9 (Suppl 1): 42.
5. Nasuti A, Sagristani M, Sessa F. Oral Sucrosomial® Iron (Sideral® Forte) is effective and well tolerated in elderly patients affected by iron deficiency anemia of various origins. Exp Rev Hematol 2016; 9 (Suppl 1): 38-39.
6. Nadir Z. Effectiveness of iron therapy with Sucrosomial® iron (Sideral® Forte) in patients with multifactorial anemia. Exp Rev Hematol 2017; 10 (Suppl 1): 37-38.
7. Svanera G. Three different conditions of iron-deficiency anemia treated with oral sucrosomial® iron therapy. Exp Rev Hematol 2017; 10 (Suppl 1): 39-40.
8. Berardi D, D'Amico F, Commatteo A, et al. Liposomial iron (Sideral®) improves fatigue in patients with myelodysplastic syndromes as refractory anemia. Multicentric study. Exp Rev Hematol 2015; 8 (Suppl 1): S26-S27.
9. Parisi S, Bruzzone M, Scarati M et al. Efficacy of Sucrosomial® Iron (Sideral® Forte) in the treatment of anemia in patients affected by systemic sclerosis. Exp Rev Hematol, 2016; 9 (Suppl 1): 21-22.
10. Bellodi A, Molinari E, Genova C, et al. Retrospective evaluation of iron deficiency patients in a Northwestern Italy anemia ambulatory: experience with Sucrosomial® Iron. Exp Rev Hematol 2016; 9 (Suppl 1): 29.
